# Early repair of open abdomen with a tailored two-component mesh and conditioning vacuum packing: a safe alternative to the planned giant ventral hernia

**DOI:** 10.1007/s10029-012-0919-0

**Published:** 2012-05-23

**Authors:** U. A. Dietz, C. Wichelmann, C. Wunder, J. Kauczok, L. Spor, A. Strauß, R. Wildenauer, C. Jurowich, C. T. Germer

**Affiliations:** 1 Department of General, Gastrointestinal, Vascular and Pediatric Surgery, University Hospital of Wuerzburg, Oberduerrbacher Strasse 6, 97080 Wuerzburg, Germany; 2 Department of Anesthesia and Critical Care, University Hospital of Wuerzburg, Oberduerrbacher Strasse 6, 97080 Wuerzburg, Germany; 3 Department of Plastic Surgery, Hand- and Burns Surgery, University of Aachen (RWTH), Pauwelsstraße 30, 52074 Aachen, Germany

**Keywords:** Giant ventral hernia, Laparostomy, Open abdomen, Vacuum conditioning, Synthetic mesh

## Abstract

**Purpose:**

Once open abdomen therapy has succeeded, the problem of closing the abdominal wall must be addressed. We present a new four-stage procedure involving the application of a two-component mesh and vacuum conditioning for abdominal wall closure of even large defects. The aim is to prevent the development of a giant ventral hernia and the eventual need for the repair of the abdominal wall.

**Methods:**

Nineteen of 62 patients treated by open abdomen over a two-year period could not receive primary abdominal wall closure. To achieve closure in these patients, we applied the following four-stage procedure: stage 1: abdominal damage control and conditioning of the abdominal wall; stage 2: attachment of a tailored two-component mesh of polyglycolic acid (PGA) and large pore polypropylene (PP) in intraperitoneal position (IPOM) plus placement of a vacuum bandage; stage 3: vacuum therapy for 3–4 weeks to allow granulation of the mesh and optimization of dermatotraction; stage 4: final skin suture. During stage 3, eligible patients were weaned from respirator and mobilized.

**Results:**

The abdominal wall gap in the 19 patients ranged in size from 240 cm^2^ to more than 900 cm^2^. An average of 3.44 vacuum dressing changes over 19 days were required to achieve 60–100 % granulation of the surface area, so final skin suture could be made. Already in stage 3, 14 patients (73.68 %) could be weaned from respirator an average of 6.78 days after placement of the two-component mesh; 6 patients (31.57 %) could be mobilized on the edge of the bed and/or to a bedside chair after an average of 13 days. No mesh-related hematomas, seromas, or intestinal fistulas were observed.

**Conclusion:**

The four-stage procedure presented here is a viable option for achieving abdominal wall closure in patients treated with open abdomen, enabling us to avoid the development of planned giant ventral hernias. It has few complications and has the special advantage of allowing mobilization of the patients before final skin closure. Long-term course in a large number of patients must still confirm this result.

## Introduction

**Fig. 1:**
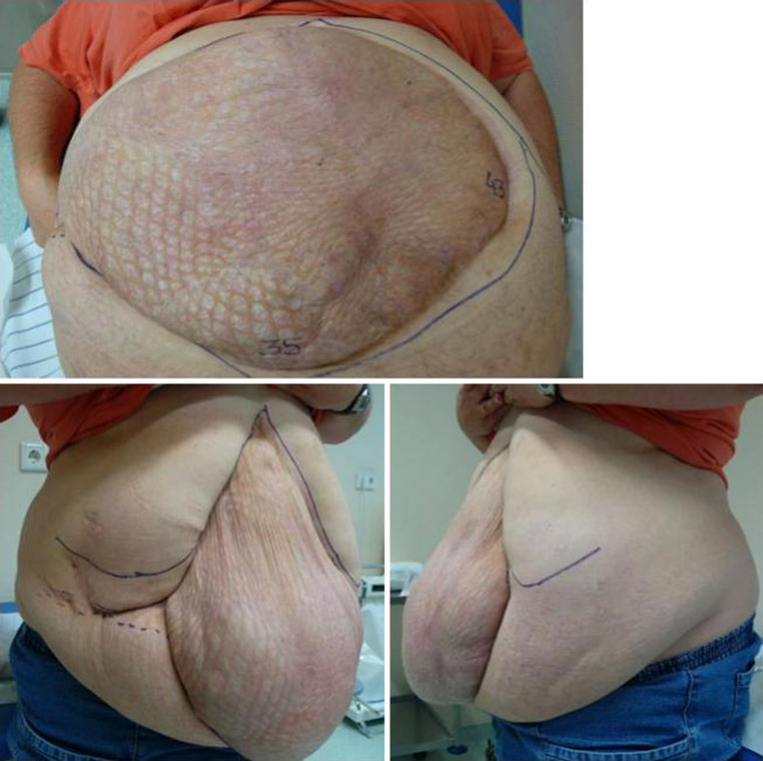
Typical planned giant ventral hernia following complicated course of a cholecystectomy in a 43-year-old female patient. The hernia begins in the medial subxiphoidal region at the costal arch and has an additional component *right lateral*. Length: 35 cm and width: 43 cm. BMI = 47; 40 pack-years

The concept of open abdomen, also termed laparostomy, was introduced in the 1970s and is widely applied today [1, 2]. In the USA, it is most often used to treat abdominal trauma, and in Germany, for secondary peritonitis. A recent poll revealed that 94 % of German clinics employ open abdomen [3]. In patients treated over several days with open abdomen, anatomic abdominal wall closure poses a challenge. Often, the fascia edges are depleted due to inflammation and retracted laterally, preventing successful abdominal wall closure. If there is sufficient granulation of the intestinal convolutions, a skin mesh cover can follow with consecutive giant ventral hernia and all the challenges associated with further abdominal wall reconstruction (Fig. 1) [1, 4–10]. Although open abdomen is a proven therapy concept, patients so treated can only gradually be mobilized during hospitalization and are subject [upon release] to major social limitations due to the grossly deformed abdominal wall. Biological meshes for septic open abdomen have still not been sufficiently tested. Experience shows however that in some cases, they are dissolved by vacuum therapy or also lysed in part by germs and that they do not prevent incisional hernias during course [11].

The concept of staged closure of open abdomen applying mesh reinforcement and coverage with whole skin is therefore attractive. Clinical experience with open abdomen patients treated with temporary absorbable meshes, Bogotá bag, and skin meshes as well as the observation that during colorectal interventions, incisional hernias could be treated with large pore polypropylene (PP) meshes without elevated risk of infection gave rise to the concept of staged therapy employing suture fixation of a mesh composed of these two components. The concept involves the following therapeutic stages: (stage 1) damage control by limiting intra-abdominal pressure and/or infection and conditioning of the abdominal wall; (stage 2) suture fixation of a two-component polyglycolic acid (PGA) and PP mesh in intraperitoneal onlay mesh position (IPOM); (stage 3) granulation-promoting vacuum conditioning of the mesh and lateral dermatotraction; and (stage 4) skin suture over the granulated mesh.

In the following, we present the surgical technique and results of this novel staged procedure that has the advantages of early patient mobilization and a low complication rate.

## Patients and methods

During a three-year period, 62 patients received three or more abdominal revisions using staged therapy. The diagnoses upon admission are listed in Table 1. The intensive care simplified acute physiology score (SAPS II) was used to assess the disease severity at admission to the intensive care unit (ICU) and again at the time of abdominal wall closure and/or suture fixation of the two-component mesh or at the time of the death [12]. Under this concept, treatment is administered in four stages.

**Table 1:** Patient characteristics

		No AW closure	Linea alba suture	Laparotomy		Total
				2-comp. mesh	Other	
Gender male/female (total)		9:6 (15)	11:10 (21)	7:12 (19)	5:2 (7)	32:30 (62)
Age Avg (SD)		60.33 (17.15)	62.25 (11.97)	60.76 (14.50)	63.85 (15.24)	n.s.
Diagnosis upon admission	Referred					
Abdom. compartment syndrome	(1)	–	–	3	1	4 (6.45 %)
Anastomotic leak small bowel	(3)	1	3	1	2	7 (11.29 %)
Anastomotic leak colon–rectum	(–)	–	–	3	1	4 (6.45 %)
Postoperative hemorrhage	(1)	–	5	–	1	6 (9.67 %)
Necrotizing pancreatitis	(1)	7	1	3	–	11 (18.33 %)
Burst abdomen	(1)	1	1	2	2	6 (9.67 %)
Primary colon perforation	(3)	1	3	1	–	5 (8.06 %)
Secondary organ perforation	(2)	1	2	3	–	6 (9.67 %)
Other	(5)	4	3	4	2	13 (20.96 %)
Total referred from other centers	17 (27.40 %)					
Number of abdominal revisions before AW closure or death: Avg (SD)		10.33 (11.10)	7.00 (3.62)	7.78 (6.42)	9.71 (6.31)	n.s.
SAPS II score						
Admission to ICU		52.00 (14.39)	54.71 (20.06)	63.80 (24.53)	n.s.	
Predicted death rate (%)		37.90 (20.03)	40.51 (28.35)	48.36 (29.74)	n.s.	
Stage 2 (AW closure or death)		67.25^a‡^ (08.77)	45.35^c^ (20.88)	44.60 (09.86)	*p* = 0.0005*	
Predicted death rate (%)		64.85^b‡^ (17.07) *p* = 0.0286*	28.40^d^ (26.12) *p* = 0.0156*	20.58 (06.52) n.s.	*p* < 0.0001*	
Mortality		15 of 15 (100 %)	7 of 21 (30.00 %)	2 of 19 (9.5 %)	1 of 7 (14.28 %)	26
Multiorg. failure, sepsis		15	7	1	1	
Pulmonary embolism		–	–	1	–	

*AW* Abdominal wall, *Avg* Average,* SD*Standard deviation,* ICU* Intensive care unit,* n.s.* not significant

^a, b^Significant increase of SAPS II score and predicted mortality rate

^c, d^Significant decrease of SAPS II score and predicted mortality rate

^‡^SAPS II score and predicted mortality rate of no AW closure >suture (*p* < 0.05) and >2-comp. (*p* < 0.0001)

**Fig. 2:**
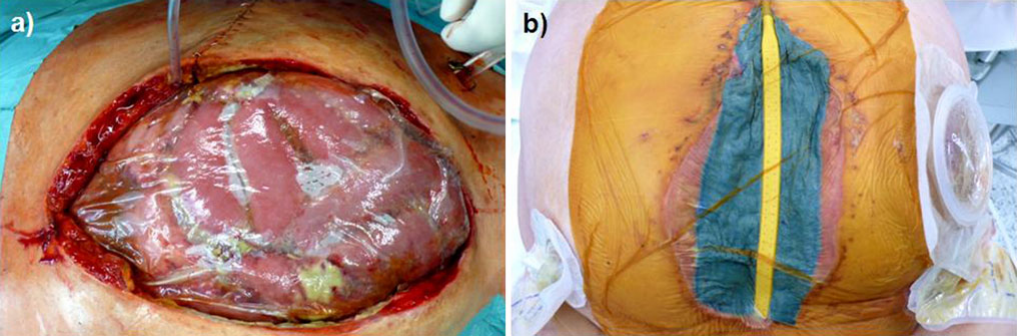
Intraoperative presentation of stage 1: **a** Insulation bag used to protect the bowels and prevent adhesion to the abdominal wall. **b** Vacuum dressing over the insulation bag, with suction drain

The goal of stage 1 is damage control, that is, control of intra-abdominal infection and pressure (Table 2). All patients underwent stage 1 treatment. During stage 1, special attention was also paid to conditioning of the abdominal wall for subsequent abdominal wall closure; any adhesions between the intestinal loops and the abdominal wall were prevented or lysed early. If a firmly adhesive intestinal convolution already existed (e.g., in patients transferred from other facilities), it was mobilized if possible in stages from the abdominal wall during revisions. As a rule, the abdominal wall should be kept free for a circumference of at least 6 cm to allow for later—during stage 2—anatomic abdominal wall closure or suture mesh fixation. To protect abdominal organs, a bandage was applied according to the technique of Barker et al. [13], using the foil-lined side of an insulation bag (Vi-Drape^®^, MCD St. Paul, MN, USA) (Fig. 2). The purpose was to prevent adhesions between the abdominal wall and the intestinal convolution and to protect the intestinal loops from excessive vacuum pressure. Macroscopic cleansing of the intra-abdominal cavity was confirmed by swabs for microbiological investigation; in the classification of Björck et al., the finding was grade 1A, 2A, or 4 [14]. The primary aim was to achieve fascial closure after stage 1. If anatomical abdominal wall closure was not possible at the end of stage 1, the patient was enrolled to abdominal wall closure through stages 2–4. This was the case in patients with rectus diastasis > 15 cm, with adhesive intestinal convolutions, and with pronounced intestinal edema.

**Table 2:** Therapeutic goals of the four-stage procedure involving application of a tailored two-component mesh and conditioning vacuum packing

Treatment stage	Goal	Criteria for the conclusion of the treatment stage
Stage 1	1. Abdominal damage control Open abdomen, Bogotá bag or;	1. Patient stabilized
		2. Peritonitis healed
	2. Relief of pressure in ACS;	3. Absence of intestinal fistulas
	3. Release of adhesions between bowel and abdominal wall	
Stage 2	1. Suture fixation of the two-component mesh to augment abdominal wall in IPOM bridging position	
	2. Application of a controlled vacuum pack	
Stage 3	[Duration: 3–4 weeks]	
	1. Vacuum conditioning to promote granulation tissue formation	1. Presence of sufficient granulation tissue Coverage of ca. 60–100 % of the mesh area with granulation tissue
	2. Staged redressing of skin (Dermatotraction)	2. Medial skin closure possible
	3. Weaning	
	4. Mobilization	
Stage 4	1. Placement of Redon drains	1. Redon drain left in place 7–10 days
	2. Secondary skin suture	2. Ultrasound of abdominal wall to exclude fluid accumulation before Redon drain removal

*ACS* abdominal compartment syndrome; *IPOM* intraperitoneal onlay mesh

**Fig. 3:**
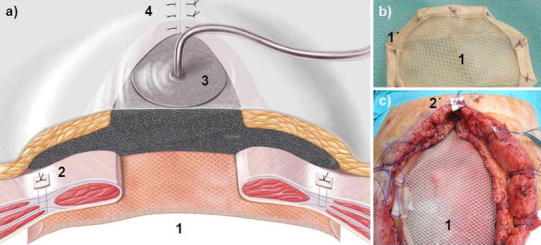
**a** Schematic presentation of stage 2; **b** the two-component mesh (*1*) with PGA hem seam (*1*′); **c** Operation site of the two-component mesh in IPOM position (*1*) as well as transfascial suture fixation with pledget (*2*′). (*1*) and (*1*′) = two-component mesh in IPOM position with the PGA layer underneath; (*2*) and (*2*′) = transfascial suture with pledget; (*3*) = Vacuum packing with polyurethane sponge tucked laterally between the fascia and subcutaneous tissue; (*4*) = Donati suture of the skin wound ending

In stage 2, a two-component mesh was fixed by suture in IPOM position and a conditioning vacuum bandage applied (Table 2). To start, subcutaneous tissue was detached from the fascia ca. 6 cm in a lateral direction. The abdominal wall defect was measured to determine the form and size of the mesh. The gap was reduced by concentric traction to ensure that the mesh would fit, the goal being to cover the facial defect with the mesh allowing a 5–6 cm overlap. The mesh was tailor-made from two different commercially available meshes: a woven PGA mesh (Dexon^®^ Style #8, Covidien, USA) attached by absorbable sutures beneath a large-pore PP mesh (Optilene^®^ Mesh Elastic, 3 mm pore size, B.Braun-Aesculap, Germany). The PGA layer is made larger in area so as to allow suture fixation around the margin of the PP mesh. To finish, PP 1 USP sutures for later transfascial fixation of the two-component mesh are placed at 5-cm intervals on the abdominal wall (Fig. 3). The two-component mesh was positioned with the PGA mesh in the IPOM position on the intestinal convolution, extended under the facial defect, and attached transfascially; if possible, the suture holes were made lateral to the rectus abdominis. To avoid tear-out, the epifascial knots were tied over a pledget (Ethisorb^®^, Ethicon, USA). For vacuum conditioning, a polyurethane sponge (KCI-Medical, USA) was tucked laterally beneath the subcutaneous tissue. The skin of the proximal and distal wound poles was closed with strong Donati sutures (e.g., with PP 2-0 USP) [15, 16]. The wound was sealed over the polyurethane sponge with adhesive foil, the “suction cup” affixed and continual vacuum therapy begun at 125 mmHg (Fig. 3).

**Fig. 4:**
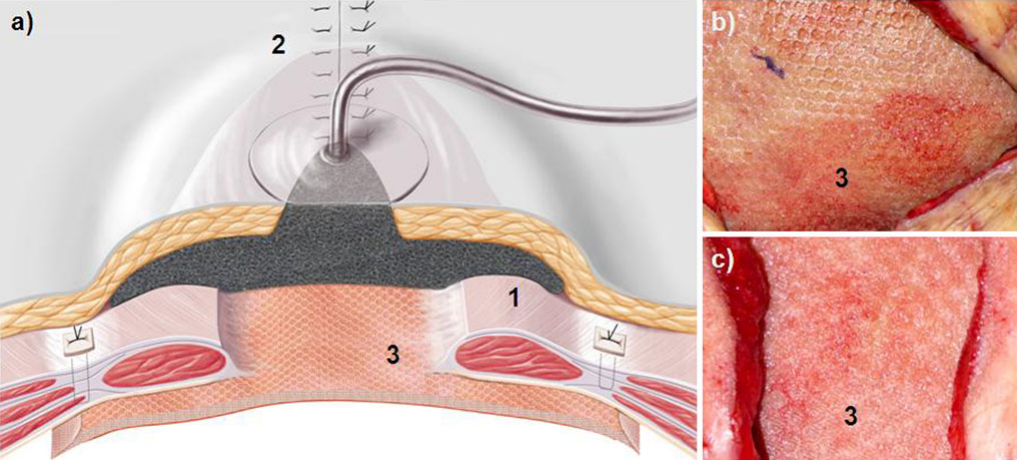
**a** Schematic presentation of stage 3; (*1*) The polyurethane sponge is trimmed under the detached subcutis but still covers the entire area of the two-component mesh; (*2*) The skin suture is optimized on the two wound poles. **a**–**c** the two-component mesh is incrementally incorporated by granulation tissue, the wound healing is uneventful (*3*); **b** 30 % granulation after 2 weeks; **c** 90 % granulation after 4 weeks

**Fig. 5:**
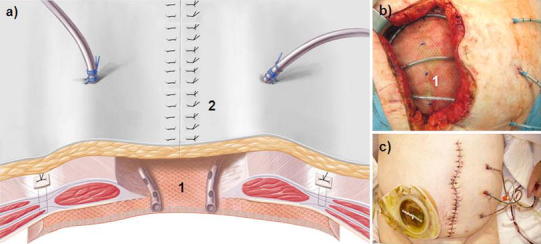
**a** Schematic presentation of stage 4; the sponge is removed, suction drains are inserted (*1*) and the overlying full thickness skin is closed (*2*); **b** site of the insertion of the suction drains running parallel to each other (*1*) and loose fascia/skin margins resulting from prior dermatotraction; **c** results on 7th postoperative day after final skin suture

Stage 3 lasted from 2 to 4 weeks and serves for wound conditioning. Its goal is to achieve granulation tissue formation in at least 50–80 % of the two-component mesh area (Table 2). The vacuum delivery volume was noted and the vacuum dressing changed every 5–6 days. At every vacuum dressing change, the skin at the proximal and distal wound poles was closed a little more and the polyurethane sponge trimmed in order to diminish the subcutaneous wound surface (Fig. 4). During stage 3, some patients could be weaned from the respirator, extubated, and mobilized to a bedside chair. Stage 3 was ended when the mesh was visibly incorporated by the granulation tissue, and the secondary skin suture of the final stage (stage 4) could be performed. Two to three suction drains running parallel to each other were placed on the granulated two-component mesh at 5-cm intervals (Table 2). To prevent dislocation of the drains, these could be attached with a 5-0 USP rapid-absorbable suture to the PP component (e.g., Safil Quick^®^ or Vicryl Rapid^®^). The four-stage procedure was concluded with subcutaneous suture and Donati skin suture with Prolene 2-0 USP (Fig. 5). The drains were left in place for prophylaxis against seroma for at least 7–10 days and removed when the delivery volume was reduced to 10 ml/24 h. Before pulling the drain, sonography of the abdominal wall was performed to exclude epifascial seroma.

**Fig. 6:**
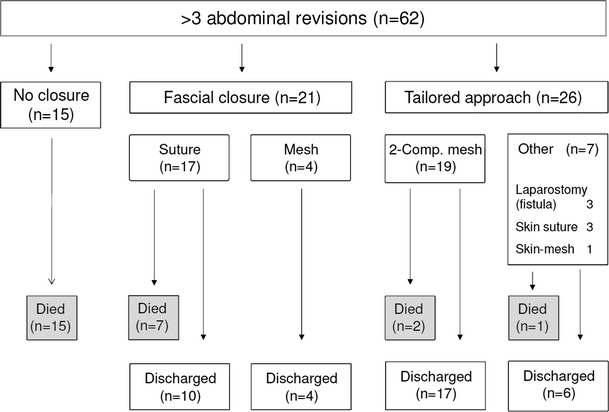
Patient flow diagram

In the group of patients with primary abdominal wall closure, the linea alba was sutured with a PDS^®^ 1 USP loop suture or reconstruction was done by implantation of a PP mesh in retromuscular sublay position with the placement of subcutaneous and/or retromuscular suction drainage for 5–6 days. In unfavorable fascial conditions, the rectus sheath layers were closed with bilateral inverted figure-eight sutures [17]. A third patient group was closed in a manner different from that described above (Table 1) (Fig. 6). These were patients with open small bowel fistulas (covered by skin mesh after the granulation of the laparostomy) or who received a secondary skin suture over the bowel convolution without fascia closure.

### Statistical analysis

Statistical analyses were carried out with GraphPad Prism^®^ and GraphPad InStat^®^ software. In univariate analysis, statistical pair analysis was used to check the relevance of the individual factors with the χ^2^ homogeneity test and/or the Fisher’s exact test: “*p*” smaller than 5 % (*p* < 0.050) was regarded as significant.

## Results

Of the 62 patients, 32 were male and 30 were female. There was no age difference between the different treatment groups. The most common surgical indication was necrotizing pancreatitis (*n* = 11), followed by small bowel anastomotic insufficiency (*n* = 7), postoperative hemorrhage, burst abdomen, and secondary organ perforation (*n* = 6 each). The number of abdominal revisions up to the conclusion of abdominal damage control (stage 1) did not differ between the treatment groups (Table 1). Fifteen patients died during stage 1 before abdominal wall closure could be realized; during intensive care involving an average of 10.33 abdominal revisions, these patients showed a significant increase in SAPS II scores of about 30 % and also of the predicted death rate of around 71 % (Table 1). In 47 patients, abdominal wall closure was achieved (Fig. 6); in this group, the SAPS II scores and predicted death rates declined significantly (Table 1). Ten of these patients died during hospitalization of multiorgan failure (*n* = 9) or pulmonary embolism (*n* = 1) (Tables 1, 3).

**Table 3:** Classification of complications following abdominal wall closure during hospitalization according to the grading system of Dindo et al. [18]

	Complication	Linea alba suture (*n* = 21)	Two-component mesh (*n* = 19)	Other (*n* = 7)
Grade I	Wound infection (conservative)	–	1	2
Grade II	MRSA colonization	–	1	–
	Pneumonia	1	1	1
Grade IIIa	CT-guided abscess drainage	–	2	1
	Sick sinus syndrome	–	1	–
Grade IIIb	Wound revision	4	–	1
	Skin necrosis	–	–	–
	Hematoma	–	–	–
	Seroma	–	–	–
	Mesh infection	–	–	–
	Stoma complication	1	–	–
	Bowel fistulas	1	–	2
Grade IV	Pulmonary embolism	–	1	–
	Renal insufficiency/dialysis	1	2	1
	Multiorgan failure	7	1	–
Grade V	Death	7	2	1
Suffix “d”	Chronic pain	–	1	–
	Chronic mesh infection	–	–	–
	Incisional hernia	3	–	6
	Readmission due to ileus	1	–	1
	CIP	1	2	1

*MRSA* methicillin-resistant Staphylococcus aureus; *CIP* critical illness polyneuropathy

Suffix “d”: the incidence of incisional hernia was diagnosed at a 1-year follow-up

In 19 patients, the four-stage abdominal wall closure with two-component mesh (Fig. 6) was performed. The mean time for abdominal damage control (stage 1) was 25 days, with a significant reduction in the SAPS II scores and predicted death rate (Table 1). The size of the abdominal wall gap as measured at the fascia edges (length vs. width) was <300 cm^2^ (240.00 ± 84.85 cm^2^) in two patients, 300–600 cm^2^ (566.66 ± 182.60 cm^2^) in eight patients, and >600 cm^2^ (867.33 ± 186.33 cm^2^) in nine patients. The average size of the implanted meshes was 644.46 cm^2^ (SD ± 291.95 cm^2^ and range 180–1,225 cm^2^). After implantation of the two-component mesh, an average of 3.44 vacuum dressing changes were necessary over a mean period of 19 days until definitive skin suture over the mesh, with 60–100 % of the implanted PP mesh incorporated by granulation. The drainage volumes of the vacuum bandage ranged from 30 to 800 ml per day (average 180 ml/24 h), depending on the mesh area and sponge size. During the vacuum conditioning phase (stage 3), weaning from the respirator could be begun in 14 patients (73.68 %) after an average of 6.78 (±7.41) days; in three patients, weaning was started even before suture fixation of the mesh. Also, in stage 3, six patients (31.57 %) began mobilization at the edge of the bed and/or to a bedside chair on average 13 days after suture fixation of the two-component mesh. Two patients (9.5 %) in this group died during hospitalization (Tables 1, 3).

In 21 patients, median abdominal wall closure was achieved by suture of the midline and skin adaptation**:** in 17 by direct suture of the linea alba and in 4 using a reinforcing PP mesh in retromuscular position (sublay). These patients were revised an average of 7 times for abdominal damage control with a significant reduction in the SAPS II scores and predicted death rates. The hospital mortality rate in this group was 30 % (*n* = 7) due to multiorgan failure (Table 1).

Seven of the 26 laparostomy patients were closed in another manner (Fig. 6); during course, these patients showed no relevant reduction in SAPS II scores and predicted death rates (Table 1). One of these 7 patients (14.28 %) died of sepsis and multiorgan failure. Three patients with open small bowel fistulas were conditioned until the fistulas matured and the granulation tissue allowed covering with a skin mesh. In another patient, the intestinal convolution was covered with a skin mesh as preferred by the surgeon. In three patients, the skin was secondarily closed over the intestinal convolution without the reconstruction of the abdominal wall (Table 1).

Abdominal wall closure was followed by different complications during hospitalization; the types of complications did not differ between the groups. It is striking that the group undergoing four-stage abdominal wall closure suffered no hematomas or seromas, and no mesh-induced intestinal fistulas. The complications are shown in Table 3 according to Dindo et al. [18].

## Discussion

Treatment for open abdomen in stages is a proven practice based on the principle that clinical problems should be divided into smaller therapeutic goals that can be addressed and solved in sequence [19]. Giant ventral hernias following treatment for open abdomen pose a challenge for patients and surgeons alike (Fig. 1). Patients with skin mesh graft-covered giant ventral hernias frequently decline delayed the reconstruction of the abdominal wall and chose instead to keep the giant hernia [20]. We describe for the first time a staged procedure for the prevention of planned giant hernias by timely mesh implantation.

The PGA mesh has been in use since 1986 to cover free lying intestinal loops. Greene et al. applied PGA meshes after debridement or partial excision of the abdominal wall, but they removed the mesh from the intestinal loop in subsequent revisions, with a consequent 13 % incidence of fistula [21]. Fabian et al. also removed the PGA mesh from intestinal convolutions prior to skin mesh coverage. Our experience indicates this would not be necessary after 3–4 weeks of conditioning [19]. In contrast to the literature, we leave the mesh in place during the entire granulation phase because the PGA layer transforms in a matter of months with neoperitoneal formation [22]. This may explain why no enteroatmospheric fistulas were seen in our patients with the PGA mesh, whereas in the studies of Jernigan et al., they arose in 8.4 % of patients after 18 days [19–21].

An essential component of most current concepts for open abdomen treatment is the application of vacuum therapy for the conditioning of the fascia, which in some cases allows median abdominal wall closure [13, 23–25]. The use of vacuum on the two-component mesh patients stabilized the abdominal organs, allowing patients mobilization without the mesh rubbing against the intestinal loops; concomitantly, the PP mesh component was progressively incorporated by the granulation tissue. In this group, weaning could begin on average 10 days, mobilization from bed 13 days after suture fixation of the two-component mesh. The complication rate was low and, most importantly, there were no wound-healing complications and no intestinal fistulas (Table 3). To extend the interval between dressing changes beyond 6 days would increase the risk of ingrowth of the sponge in the subcutaneous fat tissue and delay the goal of reduction in the subcutaneous wound area.

In a multicenter study of 151 patients, primary fascial closure was achieved with vacuum and mesh-mediated fascial traction in 76.6 % (intention to treat). Differing from our mainly septic population, in this series, the main indication for open abdomen was intra-abdominal hypertension; eight patients developed intestinal fistulas and late results regarding the incidence of incisional hernia were not reported [25]. Recently, biological meshes were introduced. The “Abdominal Ventral and Incisional Hernia Working Group” has presented an algorithm that establishes criteria for using biological meshes in accordance with the risk of infection [22]. Preliminary data however suggest that secondary healing can last several weeks at a disproportionately high cost and that in the contaminated area, there is still a risk of incisional hernia during course [11]. Synthetic meshes therefore still represent an important alternative, also in combination with absorbable meshes. The concept of two-component PGA + PP meshes derives from Afifi of Egypt, who used it in elective incisional hernia patients [26].

Regarding the surgical technique of the two-component mesh, some special aspects need to be considered. The thickness of the PGA mesh Dexon^®^ Style #8 has the advantage of allowing vacuum regulation and slow formation of granulation tissue. The suction applied to intestinal serosa promotes centrifugal growth of vessels that permeate the granulation tissue and originate in the muscularis propria of the intestine. Experience shows that thinner PGA meshes, for example Dexon^®^ Style #4, Vicryl^®^ (Ethicon, USA) or Safil^®^ (B.Braun-Aesculap, Germany)*,* are permeated more rapidly with granulation tissue. This is attributable to the stronger effect of the suction on the intestinal wall, which may increase the risk of fistula. Meshes with anti-adhesive barriers designed for the repair of incisional hernias should not be used in combination with vacuum therapy, as the resistance of the anti-adhesive barrier to the vacuum is unpredictable and impose a real risk of causing microfistulas. Since the Dexon mesh is no longer on the market, we have successfully used a double layer of Vicryl^®^ or Safil^®^ meshes in some patients. In an effort to avoid chronic pain and potential sinus fistula, transfascial sutures could be made alternatively with long-term absorbable threads, with PDS^®^ for example.

There was no correlation between the type of abdominal closure and mortality. Four patients in the group with suturing of the linea alba underwent revision due to wound-healing complications and one patient developed an intestinal fistula. The fact that we had no infection-related complications in the two-component mesh group is even more significant because the negative sequelae of mesh infection on disease course are considerable [27].The question of mesh implantation in cases of MRSA colonization is still open and must be decided on an individual basis. In some patients, long-term antibiotic therapy may be considered, although for this too no data are yet available.

The surgical technique described here cannot be generally extended for the cases of incisional hernia and in elective cases should only be performed under study conditions. Patients with incisional hernias differ from patients with open abdomen mainly in their softer intestinal wall. We successfully performed this operation in three patients with a BMI > 45, meticulously taking care that the intact greater omentum was located beneath the two-component mesh and no suction was applied directly to the intestinal loops. Despite these encouraging results, since the end of 2008, we changed our strategy in dealing with the septic abdomen to reoperations on demand and consecutively reduced the number of patients requiring this staged repair.

In summary, the four-stage procedure with the two-component mesh is a safe alternative for achieving abdominal wall closure in patients treated with open abdomen. Its complication rate is low, and it allows early mobilization and weaning from respirator. It is thus ideally suited to avoid planned giant incisional hernia with multiple interventions and to achieve satisfactory morphological and functional results. We continue to monitor these patients to ascertain the long-term follow-up, expecting an incidence of incisional hernias of about 10–20 %, depending on the individual risk factors.
